# Artificial intelligence and machine learning on diagnosis and classification of hip fracture: systematic review

**DOI:** 10.1186/s13018-022-03408-7

**Published:** 2022-12-01

**Authors:** Yonghan Cha, Jung-Taek Kim, Chan-Ho Park, Jin-Woo Kim, Sang Yeob Lee, Jun-Il Yoo

**Affiliations:** 1 Department of Orthopedic Surgery, Eulji University Hospital, Daejeon, Korea; 2 Department of Orthopedic Surgery, Ajou Medical Center, Ajou University School of Medicine, Suwon, Korea; 3 Department of Orthopedic Surgery, Yonsei 100 Percent Hospital, Incheon, Korea; 4 Department of Orthopaedic Surgery, Nowon Eulji Medical Center, Eulji University, Seoul, Korea; 5 Department of Biomedical Research Institute, Gyeongsang National University Hospital, Jinju, South Korea; 6 Department of Orthopaedic Surgery, Gyeongsang National University Hospital, 90 Chilamdong, Jinju, Gyeongnamdo 660-702 Republic of Korea

**Keywords:** Hip fracture, Artificial intelligence, Machine learning, Diagnosis, Classification

## Abstract

**Background:**

In the emergency room, clinicians spend a lot of time and are exposed to mental stress. In addition, fracture classification is important for determining the surgical method and restoring the patient's mobility. Recently, with the help of computers using artificial intelligence (AI) or machine learning (ML), diagnosis and classification of hip fractures can be performed easily and quickly. The purpose of this systematic review is to search for studies that diagnose and classify for hip fracture using AI or ML, organize the results of each study, analyze the usefulness of this technology and its future use value.

**Methods:**

PubMed Central, OVID Medline, Cochrane Collaboration Library, Web of Science, EMBASE, and AHRQ databases were searched to identify relevant studies published up to June 2022 with English language restriction. The following search terms were used [All Fields] AND (", "[MeSH Terms] OR (""[All Fields] AND "bone"[All Fields]) OR "bone fractures"[All Fields] OR "fracture"[All Fields]). The following information was extracted from the included articles: authors, publication year, study period, type of image, type of fracture, number of patient or used images, fracture classification, reference diagnosis of fracture diagnosis and classification, and augments of each studies. In addition, AI name, CNN architecture type, ROI or important region labeling, data input proportion in training/validation/test, and diagnosis accuracy/AUC, classification accuracy/AUC of each studies were also extracted.

**Results:**

In 14 finally included studies, the accuracy of diagnosis for hip fracture by AI was 79.3–98%, and the accuracy of fracture diagnosis in AI aided humans was 90.5–97.1. The accuracy of human fracture diagnosis was 77.5–93.5. AUC of fracture diagnosis by AI was 0.905–0.99. The accuracy of fracture classification by AI was 86–98.5 and AUC was 0.873–1.0. The forest plot represented that the mean AI diagnosis accuracy was 0.92, the mean AI diagnosis AUC was 0.969, the mean AI classification accuracy was 0.914, and the mean AI classification AUC was 0.933. Among the included studies, the architecture based on the GoogLeNet architectural model or the DenseNet architectural model was the most common with three each. Among the data input proportions, the study with the lowest training rate was 57%, and the study with the highest training rate was 95%. In 14 studies, 5 studies used Grad-CAM for highlight important regions.

**Conclusion:**

We expected that our study may be helpful in making judgments about the use of AI in the diagnosis and classification of hip fractures. It is clear that AI is a tool that can help medical staff reduce the time and effort required for hip fracture diagnosis with high accuracy. Further studies are needed to determine what effect this causes in actual clinical situations.

## Background

In the emergency room, clinicians spend a lot of time and are exposed to mental stress [1]. There are many things to check due to various images and laboratory tests, and fatigued clinicians (especially residents) are prone to misdiagnosis [2]. According to previous studies, it has been reported that about 2–10% of hip fractures are misdiagnosis [3]. Early diagnosis and treatment of elderly patients with hip fracture are very important for the clinical course [4]. Delay in diagnosis or surgery causes complications such as pneumonia and psoa in these patients and increases morbidity and mortality rates [1]. This not only reduces the patient's quality of life, but also causes economic exhaustion.

Diagnosis can be defined as determining the cause and characteristics of an individual patient's disease, and classification is mainly for creating a relatively homogeneous population through standardized criteria, which is mainly an important factor in disease research [5]. In addition, fracture classification is important for determining the surgical method and restoring the patient's mobility [6]. Since the surgical method is directly related to the medical cost, several countries have provided guidelines for treatment methods according to the classification of hip fractures [7]. However, classifying fractures from a lot of image information is time-consuming [8].

Currently, most medical institutions use digital medical imaging systems, which overcomes the temporal and spatial limitations of access to image information.[9] In addition, recently, with the help of computers using artificial intelligence (AI) or machine learning (ML), diagnosis and classification of hip fractures can be performed easily and quickly [10]. Studies reporting the effects of applying AI or ML to hip fracture detection used various image information such as computed tomography as well as radiographs, and presented various results on the usefulness of diagnosis and the accuracy of fracture classification.

Therefore, the purpose of this systematic review is to search for studies that diagnose and classify for hip fracture using AI or ML, organize the results of each study, analyze the usefulness of this technology and its future use value.

## Methods

### Study eligibility criteria

Studies were selected based on the following inclusion criteria: (1) studies using AI or ML techniques for diagnosis or classification of hip fracture; and (2) studies reporting on the type of imaging information used; and (3) studies reporting on statistical analysis of accuracy or area under the ROC (receiver operating characteristic) curve (AUC) for diagnosis or classification of hip fracture. Studies were excluded if they failed to meet the above criteria.

### Search methods for identification of studies

PubMed Central, OVID Medline, Cochrane Collaboration Library, Web of Science, EMBASE, and AHRQ databases were searched to identify relevant studies published up to June 2022 with English language restriction. The following search terms were used [All Fields] AND (", "[MeSH Terms] OR (""[All Fields] AND "bone"[All Fields]) OR "bone fractures"[All Fields] OR "fracture"[All Fields]). Manual search was also conducted for possibly related references. Two of us reviewed the titles, abstracts, and full texts of all potentially relevant studies independently, as recommended by the Cochrane Collaboration. Any disagreement was resolved by the third reviewer. We assessed full-text articles of the remaining studies according to the previously defined inclusion and exclusion criteria, and then selected eligible articles. The reviewers were not blinded to authors, institutions, or the publication.

### Data extraction

The following information was extracted from the included articles: authors, publication year, study period, type of image, type of fracture, number of patient or used images, fracture classification, reference diagnosis of fracture diagnosis and classification, and augments of each studies. In addition, AI name, CNN architecture type, ROI or important region labeling, data input proportion in training/validation/test, and diagnosis accuracy/AUC, classification accuracy/AUC of each studies were also extracted.

## Results

**Fig. 1:**
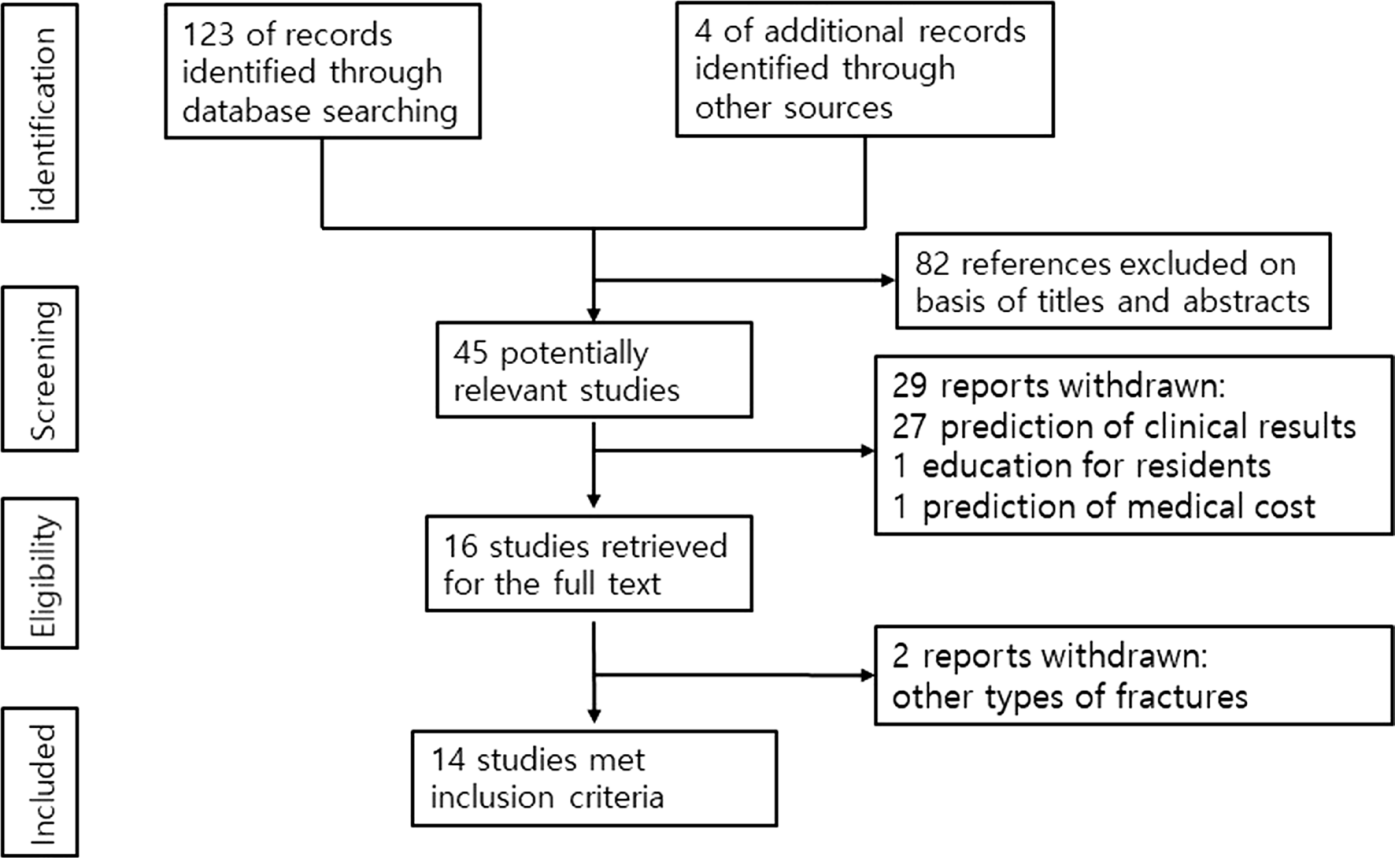
The flow chart of the study selection process

The initial search identified 123 references from the selected databases and 4 references from manual searching. Eighty-two references were excluded by screening the abstracts and titles for duplicates, unrelated articles, case reports, systematic reviews, and non-comparative studies. The remaining 45 studies underwent full-text reviews, and subsequently, 31 studies were excluded. Finally, 14 studies are included in this study [1, 7, 8, 11–21]. The details of the identification of relevant studies are shown in the flow chart of the study selection process (Fig. 1).

In 14 studies, the type of image used for AI learning was all X-ray. However, one study additionally used CT images and another additionally used CT and MRI [8, 18]. Four studies included only the neck [11, 16, 17, 21], and two studies included only the intertrochanter fracture [8, 18]. The rest of the studies included both fractures. There were 4 studies that reported the accuracy of fracture classification by AI [8, 14–16]. The number of images used varied from 234 to 10,484. The demographic data including reference diagnosis and augments method of each studies are showed in Table 1.

**Table 1:** Study, study period, demographic data of included studies

Study (Publication year)	Study period	Type of image	Type of fracture	Number of images	Fracture classification	Reference diagnosis of fracture diagnosis and classification	Augments
Adams [11]	X	X-ray	Normal, Neck Fx	805 images	Fracture (*n* = 403) No fracture (*n* = 402)	142 undergraduate students for the detection of neck of femur fractures"	MATLAB’s inbuilt image database augmentation algorithms
Urakawa [12]	2006.1–2017.7	X-ray	Normal, ITC Fx	3346 images from 1773 patients	Fracture (*n* = 1773) No fracture (*n* = 1573)	Single boardcertified orthopedic surgeon using a Digital Imaging and Communications in Medicine viewer	2650 iterations, or training of 132,500 (2650 × 50) augmented images using the adaptive moment estimation (Adam) optimizer
Cheng [13]	2008.8–2016.12	X-ray	Normal, hip Fx	3605 images	Fracture (*n* = 1975) No fracture (*n* = 1630)	The radiologist’s report, diagnosis, clinical course, and other related images, such as views of the hip joint, were reviewed	
Krogue [14]	1998–2017	X-ray	Normal, hip Fx	3026 images from 3026 patients	Nondisplaced femoral neck fractures (*n* = 182) Displaced FN fractures (*n* = 525) Intertrochanteric fractures (*n* = 765) No fracture (*n* = 1554) including Arthroplasty (*n* = 172) and Open reduction internal fixation (*n* = 59)	Two postgraduate year 4 orthopedic residents using the Visual Geometry Group Image Annotator	Three types of contrast changing cut-out, Gaussian-mixture masking, and bounding box wiggling
Yu [15]	X	X-ray	Normal, hip Fx	1061 images from 617 patients	Subcapital or transcervical fracture (*n* = 185) Basicervical or intertrochanteric fracture (*n* = 216) Subtrochanteric fractures (*n* = 50) No fracture (*n* = 610)	Local experts	X
Mutasa [16]	2000.2–2017.2	X-ray	Normal, Neck Fx	1063 images from 550 patients	Garden I/II fracture (*n* = 127) Garden III/IV fracture (*n* = 610) No fracture (*n* = 326)	Musculoskeletal fellowship-trained radiologists	(1) 1063 Source images modification including image flipping, random rotation, and random contrast jittering (2) 6000 digitally reconstructed radiographs (DRRs) were generated using simulated X-ray volume rendering (3) Additional 2000 training examples were generated utilizing a generative adversarial network (GAN)
Beyaz [17]	2013.1–2018.1	X-ray	Normal, Neck Fx	234 images from 65 patients	Fracture (*n* = 149) No fracture (*n* = 85)	X	2106 Source images modification including rotation, and Gaussian noise
Mawatari [18]	2004.4–2018.4	X-ray, CT, MRI	Normal, hip Fx	352 images	Fracture (*n* = 327) No fracture (n = 25)	2 radiologists	Image rotation with the allowance of plus and minus one degree, and horizontal flipping. (n = 3300)
Yamada [19]	2014.3–2020.1	X-ray	Normal, hip Fx	2,923 images (1,703 anteriposterior view, 1220 lateral view) from 1035 patients	Fracture (*n* = 1983) No fracture (*n* = 940)	2 board-certified orthopedic surgeons	Rotation angle range of 20°, width shift range of 0.2, height shift range of 0.2, brightness range of 0.3–1.0, and a horizontal flip of 50% using ImageDataGenerator
Cheng [20]	2008.8–2016.12	X-ray	Normal, hip Fx	3605 images, 587 Real data	Fracture (*n* = 1975) No fracture (*n* = 1630)	Based on all the available clinical information, including clinical diagnosis, imaging reports, advanced imaging reports, and operative findings"	X
Yoon [8]	2016–2018	X-ray, CT	Normal, ITC Fx	3343 images	Two classes: no fracture and fracture Three classes: no fracture, A1.1 to A2.1, and A2.2 to A3.3 Four classes: no fracture, A1, A2, and A3 Seven classes: no fracture, each type of A1.1 to A1.3, each type of A2.1 to A2.3, and A3 Ten classes: no fracture, each type of A1.1 to A1.3, each type of A2.1 to A2.3, and each type of A3.1 to A3.3 Each classes are according to AO/OTA classification [1]	Orthopedic surgeons	X
Sato [1]	2009–2019	X-ray	Normal, hip Fx	10,484 images from 4,851 patients	Fracture (*n* = 5242) No fracture (*n* = 4851)	2 orthopedic surgeons	X
Bae [21]	2005.1–2018.12	X-ray	Normal, Neck Fx	4189 images	Fracture (*n* = 1109) No fracture (*n* = 3080)	2 emergency medicine specialists	Random transformation including flip, flop, or rotation
Murphy [7]	X	X-ray	Normal, hip Fx	3659 images	Intracapsular fracture (*n* = 1082) Trochanteric fracture (*n* = 974) No fracture (*n* = 1603)	2 musculoskeletal experts (consultant orthopedic surgeon and/or consultant musculoskeletal radiologist)	Random rotating the images (−10° and 10°), grayscale-inverted (chosen at random), and using mirrored images. (*n* = 47,698)

*Fx.* fracture, *ITC* intertrochanter, *CT* computed tomography, *MRI* magnetic resonance image, *AI* artificial intelligence, *AUC* area under the ROC curve, *ROC* receiver operating characteristic

**Fig. 2:**
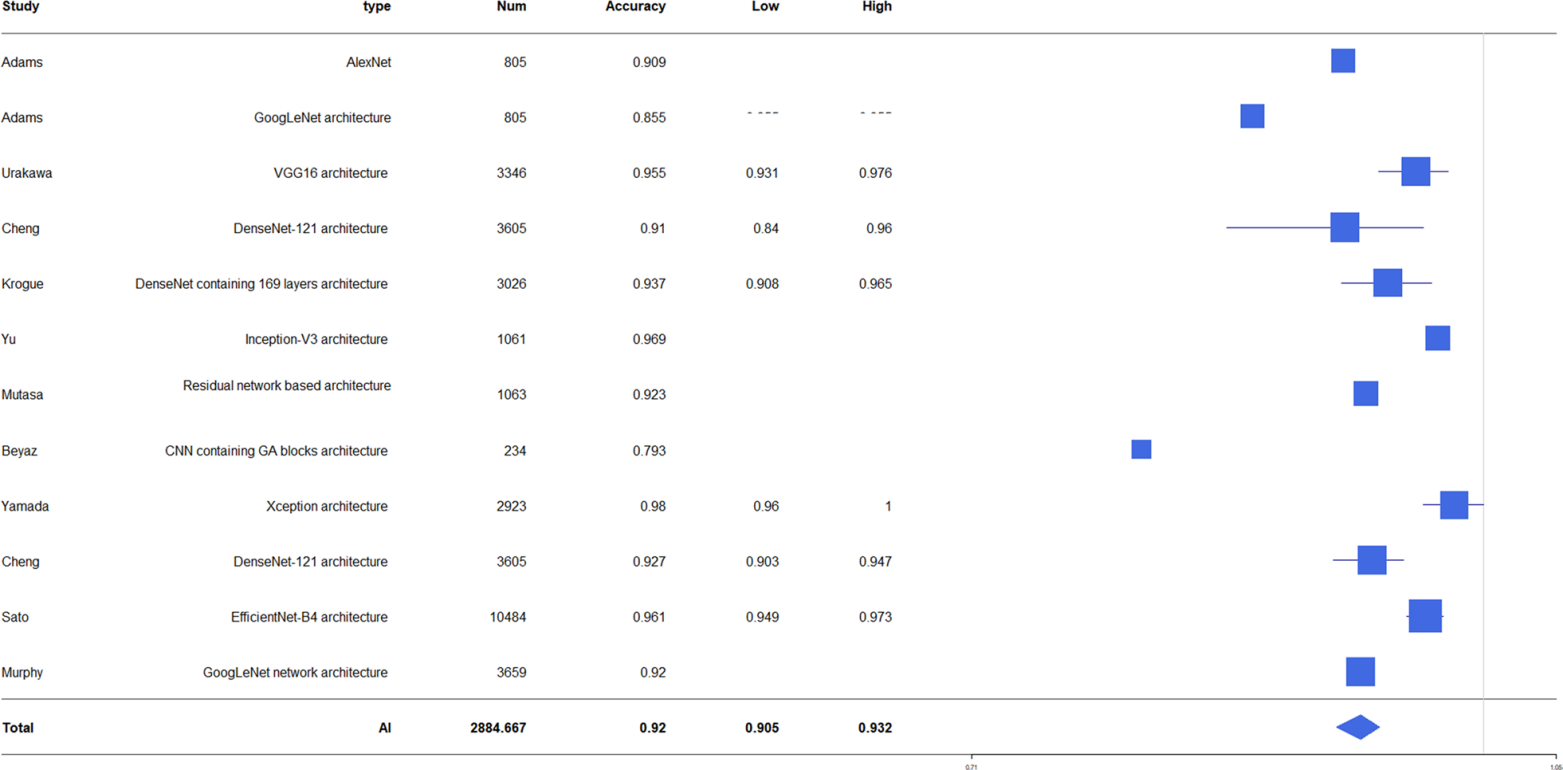
Forest plot of artificial intelligence (AI) diagnosis accuracy

**Fig. 3:**
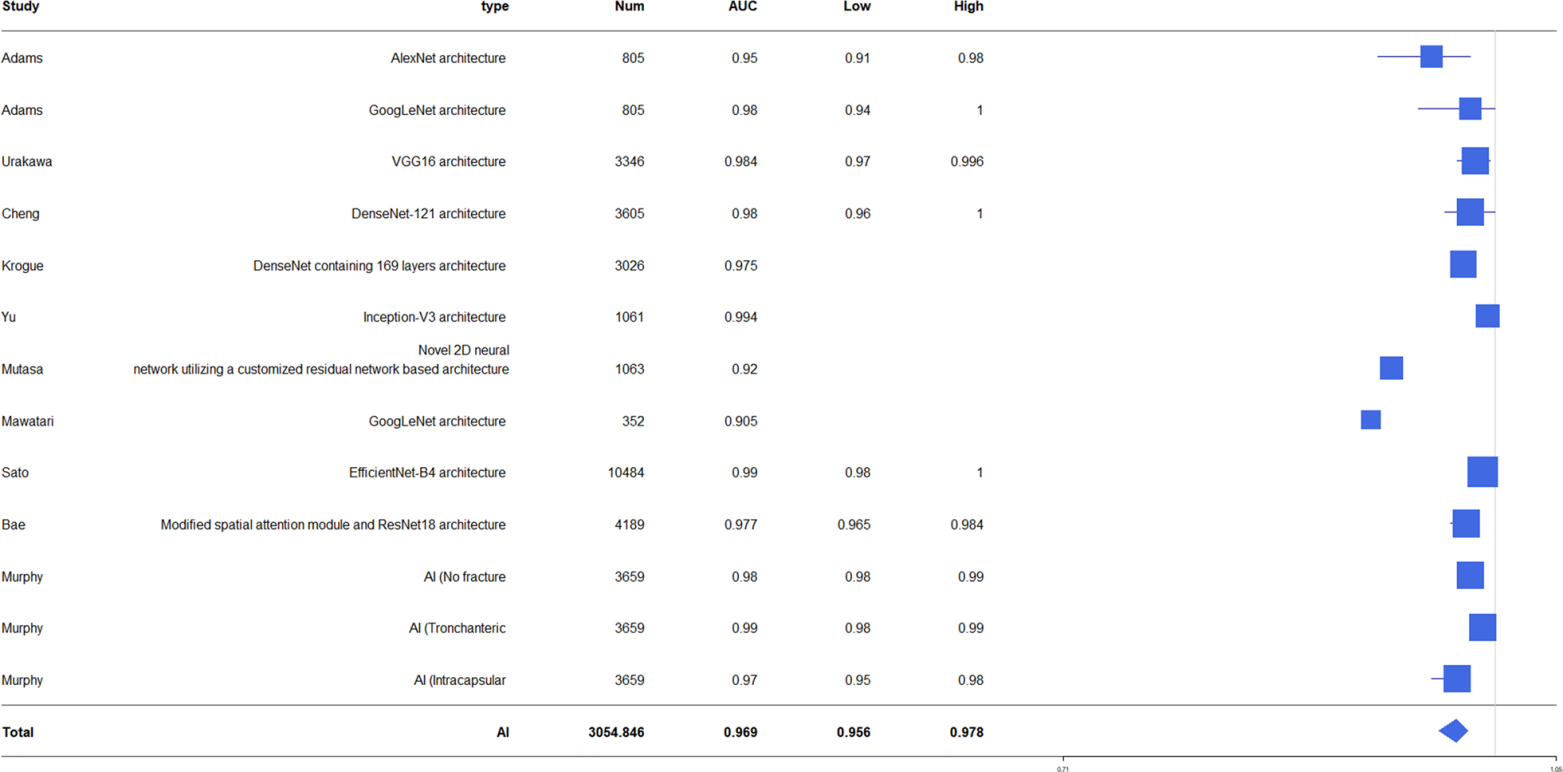
Forest plot of AI diagnosis area under the curve (AUC)

**Fig. 4:**
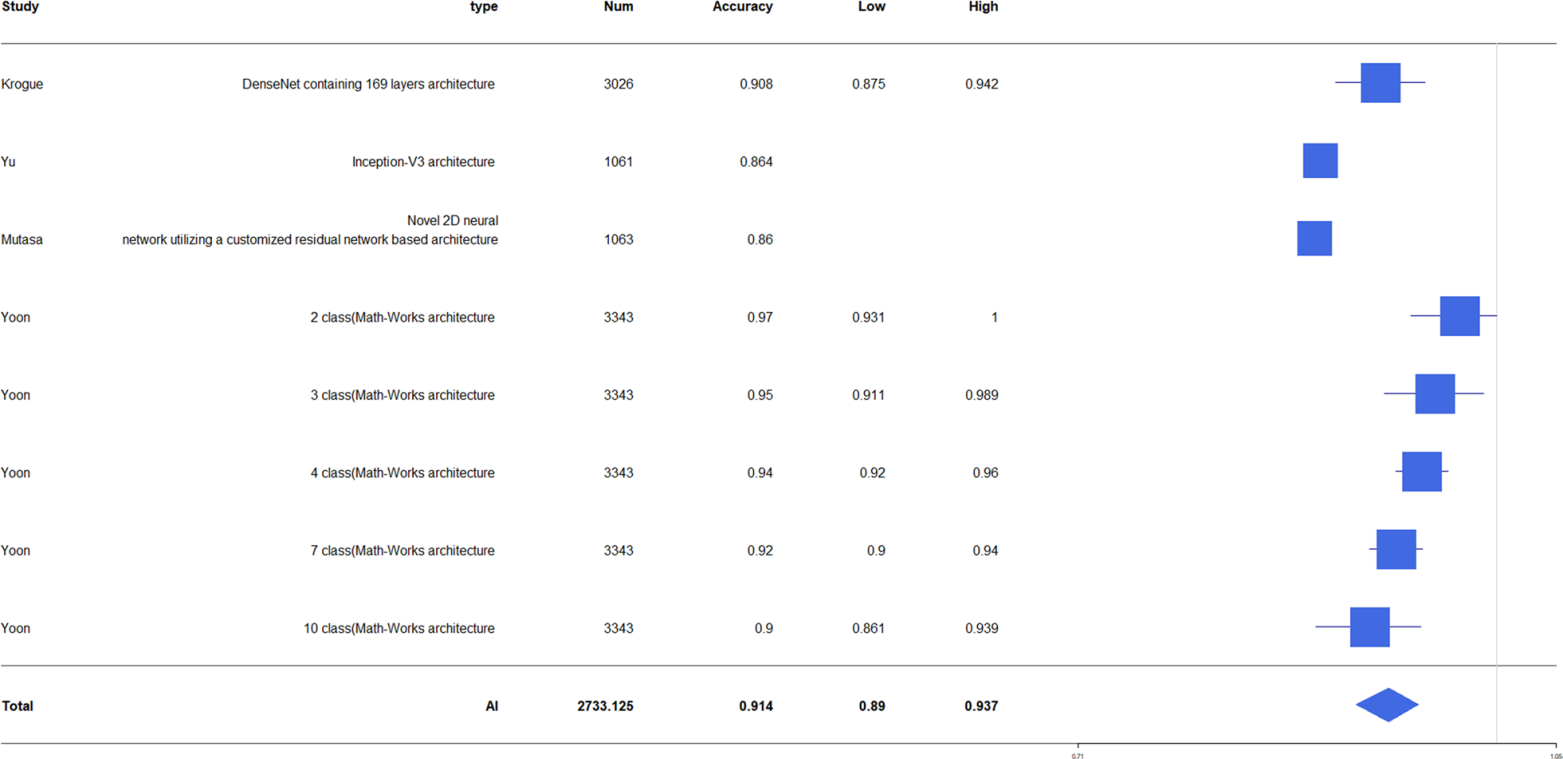
Forest plot of AI classification accuracy

**Fig. 5:**
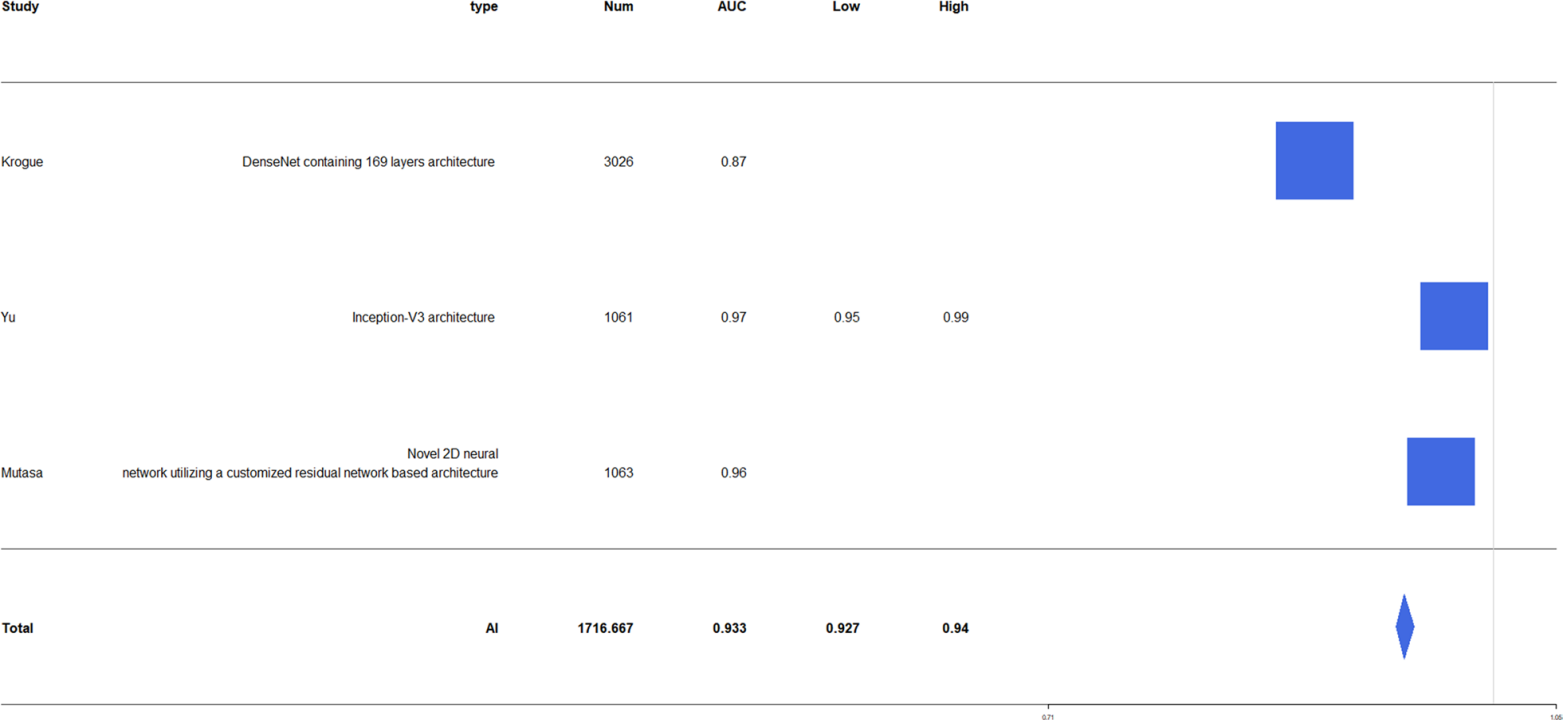
Forest plot of AI classification AUC

The accuracy of diagnosis for hip fracture by AI was 79.3–98%, and the accuracy of fracture diagnosis in AI aided humans was 90.5–97.1. The accuracy of human fracture diagnosis was 77.5–93.5. AUC of fracture diagnosis by AI was 0.905–0.99. The accuracy of fracture classification by AI was 86–98.5 and AUC was 0.873–1.0 (Table 2). The forest plot of AI accuracy and AUC of diagnosis and classification is presented in Figs. 2, 3, 4, 5. In the included study, the mean AI diagnosis accuracy was 0.92 (Fig. 2), the mean AI diagnosis AUC was 0.969 (Fig. 3), the mean AI classification accuracy was 0.914 (Fig. 4), and the mean AI classification AUC was 0.933 (Fig. 5).

**Table 2:** Accuracy and AUC of fracture diagnosis and fracture classification in included studies

Study	Fx. Diagnosis		Fx. classification	
	Accuracy (%)	AUC	Accuracy (%)	AUC
Adams [11]	88.1–94.4 (AI) 93.5 (specialist) 92.9 (residents) 90.5 (AI + medically naïve) 87.6 (medically naïve)	0.94–0.98 (AI)		
Urakawa [12]	95.5 (AI) 92.2 (human)	0.984 (AI) 0.969 (human)		
Cheng [13]	91 (AI)	0.98 (AI)		
Krogue [14]	93.7 (AI)	0.975 (AI)	91.2 (AI)	0.873–1.00 (AI)
Yu [15]	96.9 (AI)	0.9944 (AI)	93.9–98.5 (AI)	0.95–0.99 (AI)
Mutasa [16]	92.3 (AI)	0.92 (AI)	86 (AI)	0.96 (AI)
Beyaz [17]	79.3 (AI)			
Mawatari [18]		0.832 (human) 0.905 (AI) 0.876 (AI + human)		
Yamada [19]	98 (AI)			
Cheng [10]	92.67(AI) 97.1 (AI + human)			
Yoon [8]	97 (AI)		90 (AI)	
Sato [1]	96.1 (AI) 84.7 (human) 91.2 (AI + human)	0.99(AI)		
Bae [21]	97.1 (AI)	0.977 (AI)		
Murphy [7]	77.5 (human) 92 (AI)	0.98 (AI) for normal 0.99 (AI) for neck Fx 0.97(AI) for ITC Fx		

*Fx* fracture, *AI* artificial intelligence, *AUC* area under the ROC curve, *ROC* receiver operating characteristic, AI + human: AI aided human

Among the included studies, the architecture based on the GoogLeNet architectural model [7, 11, 18] or the DenseNet architectural model [13, 14, 20] was the most common with three each. Among the data input proportions, the study of Adams et al. had the lowest training rate of 57% [11], and the study of Yamada et al. had the largest training rate of 95% [19]. In 14 studies, 5 studies used Grad-CAM for highlight important regions. The information on AI for all included studies is presented in Table 3 [1, 8, 16, 20, 21].

**Table 3:** AI of included studies

Study (Publication year)	AI name	CNN architecture type	ROI or Important region labeling	Data input proportion in training/validation/test
Adams [11]	DCNN	AlexNet or GoogLeNet architectural model	X	Training-57% Validation-29% Test-14%
Urakawa [12]	CNN	VGG16 architectural model	X	Training-80% Validation-10% Test-10%
Cheng [13]	DCNN	DenseNet-121 architectural model	Image labeling and preprocessing = Each image was reviewed by a trauma surgeon for the preciseness of the label and quality of the images	Training-60% Validation-20%
Krogue [14]	Deep learning model	DenseNet containing 169 layers architectural model	Object detection algorithm to place the bounding boxes automatically = Single-shot detector with the Resnet-50 feature pyramid network architecture	Training-60% Validation-25% Test-15%
Yu [15]	CNN	Inception-V3 architectural model	RoI identifying = Each ROI was either approved or revised by the local expert	Training-60% Validation-20% Test-20%
Mutasa [16]	CNN	Novel 2D neural network utilizing a customized residual network based architecture	Highlight important regions = Gradient-weighted class activation mapping (Grad-CAM)	Training & validation-90% Test-10%
Beyaz [17]	CNN	CNN containing GA blocks architectural model	Highlight important regions = Regions containing both fractured and non-fractured femoral necks were cropped from the X-ray images manually	X
Mawatari [18]	DCNN	GoogLeNet architectural model	RoI identifying = All radiographs were manually checked and annotated retrospectively by the three radiologists referring to CT and MRI for RoI selection	Training-85% Test-15%
Yamada [19]	CNN	Xception architectural model	Highlight important regions = Orthopedic surgeon (3 years of experience) performed the image preprocessing using Paint 3D (Microsoft Corp, Redmond, WA, USA) by cropping the minimum region containing the femoral head and greater and lesser trochanters	Training-95% Validation-5%
Cheng [20]	DCNN	DenseNet-121 architectural model	Highlight important regions = Grad-CAM	Training-60% Validation-20% Test-20%
Yoon [8]	Deep faster R-CNN	Math-Works (VGG-16 architecture) architectural model	Highlight important regions = Grad-CAM	Training-80% Test-20%
Sato [1]	CNN	EfficientNet-B4 architectural model	Highlight important regions = Grad-CAM	Training-80% Validation-10% Test-10%
Bae [21]	CNN	Modified spatial attention module (CBAM + +) and ResNet18 architectural model	Highlight important regions = Grad-CAM	Training-80% Validation-10% Test-10%
Murphy [7]	CNN1 and CNN2	GoogLeNet architectural model	RoI identifying = MATLAB Training Image Labeller Application (tool)	Training-60% Validation-20% Test-20%

*AI* Artificial Intelligence, *CNN* Convolutional Neural Networks, *DCNN* Deep convolutional neural network, *GA* Genetic Algorithms, *RoI* Region of Interest, *Grad-CAM* Gradient-weighted class activation mapping

## Discussions

### Expected effects of AI in hip fracture diagnosis

As human lifespans prolong and the elderly population grows, the socioeconomic problems associated with hip fractures and postoperative care are public concerns worldwide [13]. Early diagnosis and treatment are essential to preserving patient function, improving quality of life and alleviating economic burden. Rapid diagnosis of non-displaced hip fractures by human could be difficult and sometimes requires the use of additional radiographs, bone scans, CT, or MRI. But, these additional tests are not always available in all hospitals. In addition, demineralization and overlying soft tissues may interfere with diagnosis of hip fracture [18]. Delayed diagnosis and treatment may lead to complications, such as malunion, osteonecrosis, and arthritis [19]. Moreover, as total number of imaging and radiological examinations has increased, radiology departments cannot report all acquired radiographs in timely manner [7]. For this reason, several studies on detecting hip fractures using ML have already been reported [1, 7, 8, 11–21]. Early diagnosis of hip fracture by AI algorithm in clinical course could help reduce medical costs, facilitate further preventive practices, and increase the quality of health care [20]. It also improves the allocation of resources, reduce the need for unnecessary consultations, and facilitate faster patient disposition. In particular, physicians can focus on conceptually more demanding tasks in high-volume clinics. However, reports on the effectiveness of early diagnosis of hip fractures by AI algorithm seem to be insufficient. It is considered that further studies are needed.

### CNN architecture used for hip fracture diagnosis

In this study, several CNN structures were used for radiograph image analysis in each study for hip fracture diagnosis. Among the included studies, CNNs using DenseNet or GoogLeNet architecture models were used the most. These two CNNs are inception architecture, which are deep CNNs with an architecture design composed of repeating components [22]. GoogLenet is a CNN architecture with 22 layers and is widely used in image analysis such as radiographs because of its excellent ability to recognize visual patterns [23]. In addition, GoogLeNet has 9 inception modules including 1 × 1 convolution which allows to derive various characteristics by accumulating the feature maps generated in the previous layer [22]. This structure of GoogLenet allows to extract features from different layers without the need for additional computational burdens [24]. DenseNet is a Dense Convolution Network, a CNN that can receive input from all previous layers through concatenation in a more advanced architecture than that of GoogleNet. DenseNet has the advantage of increasing computational efficiency through a compact network and being able to train by considering more diverse feature sets in all layers [25]. In addition, Inception-V3 and Xception used in the included studies are the more advanced CNN architectures of GoogLenet. These results suggested that researchers have been applied progressively advanced CNN architectures of AI for hip fracture diagnosis (Table 3).

### Diagnosis accuracy in AI versus human: Can AI replace human role in hip fracture diagnosis?

In the results of the articles included in our study, the accuracy of diagnosis for hip fracture by AI algorithm was over 90%, except for the results of Beyaz et al., and AUC of fracture diagnosis was over 0.9, which was very high [17]. Also, the diagnostic accuracy of AI was higher in a comparative study on the accuracy of hip fracture diagnosis between AI and human. Urakawa et al. presented a AI model that detected intertrochanteric fractures with an accuracy of 95.5% and an AUC of 0.984 [12]. This was higher than human's diagnostic accuracy of 92.2% and AUC of 0.969. Adams et al. reported a conventional neural network model to diagnose femoral neck fractures with an accuracy of 88.1–94.4% [11]. These figure is also comparable to experts and resident`s diagnostic accuracy of 93.5 and 92.9%. In the study of Cheng et al. and Sato et al., human diagnostic accuracy was lower than that by AI algorithm [1, 20]. Nevertheless, it is still questionable whether can AI replace human role in hip fracture diagnosis. Bae et al. used AI to diagnose femoral neck fracture after deep learning of AI using 4,189 images. Diagnostic accuracy of AI algorithm was 97.1%. However, they reported that it is difficult to detect a non-displaced fracture of the femoral neck, despite high diagnostic accuracy of AI [21]. This means that AI can reveal the limits of diagnosis in cases where AI is not trained or lacks learning. In addition, since all AI systems included in this study are not integrated with other clinical information, we consider that the clinical suspicion of human for occult fracture through evaluation of the patient's overall condition cannot yet be simulated by AI algorithm. Mawatari et al. also argued that, because the AUC values of AI aided experts were higher than the AI algorithm alone, a valid diagnosis could not be obtained by the radiograph alone, and it was inevitably affected by the quality of AI algorithm [18]. Thus, we believed that AI algorithm does not totally replace human intelligence in the current clinical environment; however, AI algorithms can complement and augment the ability and knowledge of physicians.

The increase in human dependence on hip fracture detection using AI algorithm may be another issue because it is difficult and time-consuming for doctors to make their own clinical judgments by synthesizing the results of examinations performed face-to-face with patients [20]. To solve this issue, Cheng et al. made the hip fracture detection site by AI to be highlighted and displayed so that physicians could check the results of the AL algorithm and make a final clinical judgment [20]. With the development of technology, the AI algorithm will further develop, and the tendency of humans to rely on AI will increase further in future. Further research is needed for further solutions to this problem in future.

### Efforts for AI deep learning and high diagnostic accuracy for hip fracture

Because deep learning of AI automatically and adaptively learn features from data, large and clean datasets are required [17]. Better results for detection of hip fracture by AI are decided according to the number of images. In our study, we summarized the 2 methods suggested by previous studies to overcome this. The first is data augmentation and generation where data are manipulated to artificially enlarge the dataset. The number of patients visiting a single hospital is limited, and acquiring image information from other institutions may cause a problem of personal information leakage. Sato et al. created augmented 10,484 images by classifying the images of 4851 patients into fractured side and normal side according to the time they were taken, and used it for deep learning of AI [1]. Mutasa et al. created 9063 augmented images with 737 hip fracture images and 326 normal images in 550 patients, and Beyaz et al. also generated 2106 augmented images from 234 radiographs of 65 patients [16, 17]. The second is to use various type of image information. Yu et al. reported that a distinctive fracture line or cortical angular deformity of a neck fracture is easy to detect in a single radiographic view, but a larger sample size is required for intertrochanteric fractures with complex and multiple fracture lines because the spectrum of fracture morphology is large [15]. Also, soft tissue shading or femur alignment variation may affect the detection of fractures by AI [13]. To overcome this, Yamada et al. argued that the fracture detection rate could be increased by adding a lateral view as well as a hip AP view [19]. On the other hand, Yoon et al. reported that CT images as well as radiographs were used for fracture classification of intertrochanteric fractures, reducing time consumption due to fracture classification and helping to plan accurate surgery [8]. Also, Mawatari et al. used MRI as well as CT for hip fracture detection [18]. However, this has a disadvantage in that additional cost is consumed and it is difficult to obtain a normal hip lateral view.

As AI can quickly process large amounts of patient information, it has incredible potential in diagnosing and classifying patients' diseases [26]. Especially the usefulness of AI is being studied in the trauma prediction, which has a wide range of individual differences in the number and severity of injuries due to the involvement of many external and internal factors [27]. The present study is expected to be helpful in verifying the effectiveness of AI in diagnosing these specific diseases.

There are several limitations in our study. First, we did not consider the type of AI algorism and degree of training of AI algorism. Second, we did not consider the quality of radiographs for deep learning. The selected images are likely to have high quality. Also, these images can only represent characteristics of a specific age and sex. Third, implants used for surgical treatment of hip fracture were not considered.

## Conclusions

We expected that our study may be helpful in making judgments about the use of AI in the diagnosis and classification of hip fractures. It is clear that AI is a tool that can help medical staff reduce the time and effort required for hip fracture diagnosis. Further studies are needed to determine what effect this causes in actual clinical situations.

## Data Availability

All data generated or analyzed during this study are included in this published article.
